# Defective Mitochondrial Cardiolipin Remodeling Dampens HIF-1α Expression in Hypoxia

**DOI:** 10.1016/j.celrep.2018.09.057

**Published:** 2018-10-16

**Authors:** Arpita Chowdhury, Abhishek Aich, Gaurav Jain, Katharina Wozny, Christian Lüchtenborg, Magnus Hartmann, Olaf Bernhard, Martina Balleiniger, Ezzaldin Ahmed Alfar, Anke Zieseniss, Karl Toischer, Kaomei Guan, Silvio O. Rizzoli, Britta Brügger, Andrè Fischer, Dörthe M. Katschinski, Peter Rehling, Jan Dudek

**Affiliations:** 1 Department of Cellular Biochemistry, University Medical Center Göttingen, GZMB, 37073 Göttingen, Germany; 2 Department for Epigenetics and Systems Medicine in Neurodegenerative Diseases, German Center for Neurodegenerative Diseases (DZNE) Göttingen, Göttingen, Germany; 3 Heidelberg University Biochemistry Center (BZH), University of Heidelberg, Heidelberg 69120, Germany; 4 Institute of Pharmacology and Toxicology, Technische Universität Dresden, Dresden, Germany; 5 Institute of Cardiovascular Physiology, University Medical Center Göttingen, Göttingen, Germany; 6 Department of Cardiology and Pneumology, University Medical Center Göttingen, Göttingen, Germany; 7 German Center for Cardiovascular Research (DZHK), Göttingen, Germany; 8 Department of Neuro- and Sensory Physiology, University Medical Center Göttingen, Göttingen, Germany; 9 Department of Psychiatry and Psychotherapy, University Medical Center Göttingen, Göttingen, Germany; 10 Max Planck Institute for Biophysical Chemistry, 37073, Göttingen, Germany

**Keywords:** NF-κB signaling, Hif1 alpha, mitochondria, ROS, cardiolipin, lipid, Barth syndrome, respiratory chain

## Abstract

Mitochondria fulfill vital metabolic functions and act as crucial cellular signaling hubs, integrating their metabolic status into the cellular context. Here, we show that defective cardiolipin remodeling, upon loss of the cardiolipin acyl transferase tafazzin, decreases HIF-1α signaling in hypoxia. Tafazzin deficiency does not affect posttranslational HIF-1α regulation but rather HIF-1α gene expression, a dysfunction recapitulated in iPSC-derived cardiomyocytes from Barth syndrome patients with tafazzin deficiency. RNA-seq analyses confirmed drastically altered signaling in tafazzin mutant cells. In hypoxia, tafazzin-deficient cells display reduced production of reactive oxygen species (ROS) perturbing NF-κB activation and concomitantly HIF-1α gene expression. Tafazzin-deficient mice hearts display reduced HIF-1α levels and undergo maladaptive hypertrophy with heart failure in response to pressure overload challenge. We conclude that defective mitochondrial cardiolipin remodeling dampens HIF-1α signaling due to a lack of NF-κB activation through reduced mitochondrial ROS production, decreasing HIF-1α transcription.

## Highlights

• Defective remodeling of mitochondrial cardiolipin dampens HIF-1α signaling
• ROS-mediated NF-κB activation is impaired in cardiolipin-deficient cells
• Defective NF-κB-mediated HIF-1α gene induction decreases the cellular response to hypoxia
• Deregulated cardiac response to pressure overload in Barth syndrome mouse

## Graphical Abstract

**Figure:**
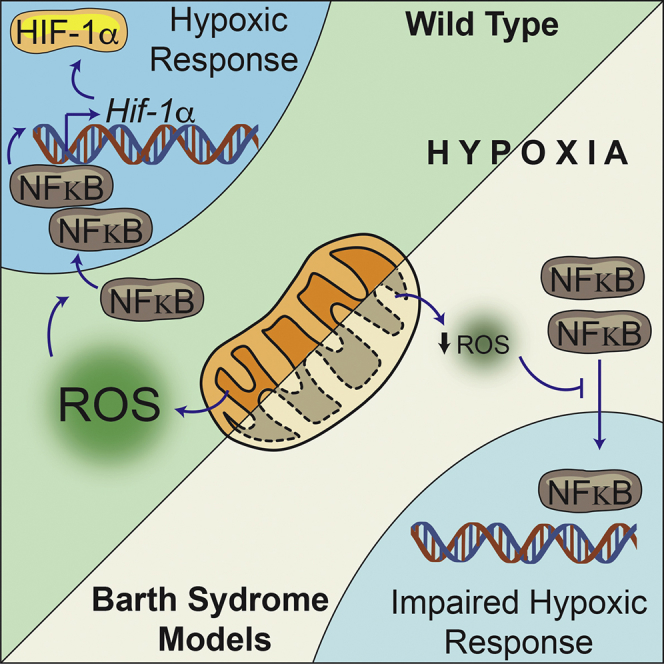

## Teaser

Defective remodeling of the mitochondrial phospholipid cardiolipin causes cardiomyopathy in Barth syndrome patients. Chowdhury et al. show that dysfunctional mitochondria affect the HIF-1α response to hypoxia. They demonstrate that mitochondrial ROS is required for NF-κB-mediated gene induction of HIF-1α.

## Introduction

Mitochondrial functions are governed by alterations in nuclear gene expression, adapting them to cellular demands. Under conditions of low oxygen tension, the respiratory chain undergoes a dynamic exchange of selected structural subunits that is governed by altered gene expression initiated by the transcription factor HIF-1 (Fukuda et al., 2007). The cellular abundance of HIF-1α is regulated by an oxygen-dependent hydroxylation process, catalyzed by prolyl-4-hydroxylase domain enzymes (PHD1–3). These enzymes target HIF-1α to proteasomal degradation. In hypoxia, diminished hydroxylation and concomitant inhibition of turnover stabilize HIF-1α, leading to increased protein amounts. HIF-1 is a master transcriptional regulator that adapts cellular metabolism to conditions of low oxygen, thereby stimulating glycolytic metabolism and altering the composition of mitochondrial oxidative phosphorylation complexes in the inner membrane. In addition to the composition of individual complexes, the organization of the respiratory chain into supercomplexes has been found to dynamically respond to physiological challenges, providing another regulatory level (Garaude et al., 2016, Greggio et al., 2017). Moreover, the activity of individual complexes can be modulated by phosphorylation, allosteric interactions, and exchange of individual subunits in response to oxygen-sensitive gene expression processes that depend on HIF-1 activity, as described above (Acín-Pérez et al., 2011, Napiwotzki et al., 1997).

The regulation of mitochondrial metabolism is critical for cellular functions and differs significantly between tissues and environmental conditions. In the case of the heart, mitochondria provide 90% of the energy demand by oxidative phosphorylation (Ventura-Clapier et al., 2004). Accordingly, defects in mitochondrial functions are frequently linked to human disorders. One example is Barth syndrome, which represents a severe X-linked cardiomyopathy caused by mutations in the TAZ gene, encoding the mitochondrial transacylase tafazzin (Bione et al., 1996). Tafazzin facilitates cardiolipin remodeling, converting monolysocardiolipin to a mature cardiolipin molecule with distinct unsaturated fatty acid side chain composition. This altered lipid composition affects inner membrane morphology as well as activity and organization of the oxidative phosphorylation system. Because respiratory chain supercomplexes, formed by complexes I, III, and IV (Wu et al., 2016), are structurally dependent on interactions with cardiolipin, they are destabilized in Barth syndrome models (Dudek et al., 2013, Dudek et al., 2016, Pfeiffer et al., 2003).

To this end, anterograde signaling processes that adapt mitochondrial function through proteomic adaptation are well established. However, it is still ill defined as to how mitochondria are able to affect nuclear gene expression upon an altered metabolic state (e.g., in the case of changes in the oxidative phosphorylation capacity, in a retrograde manner to adapt nuclear gene expression).

## Results and Discussion

### Tafazzin-Deficient Cells Display Reduced Levels and Activity of HIF-1α

**Figure 1:**
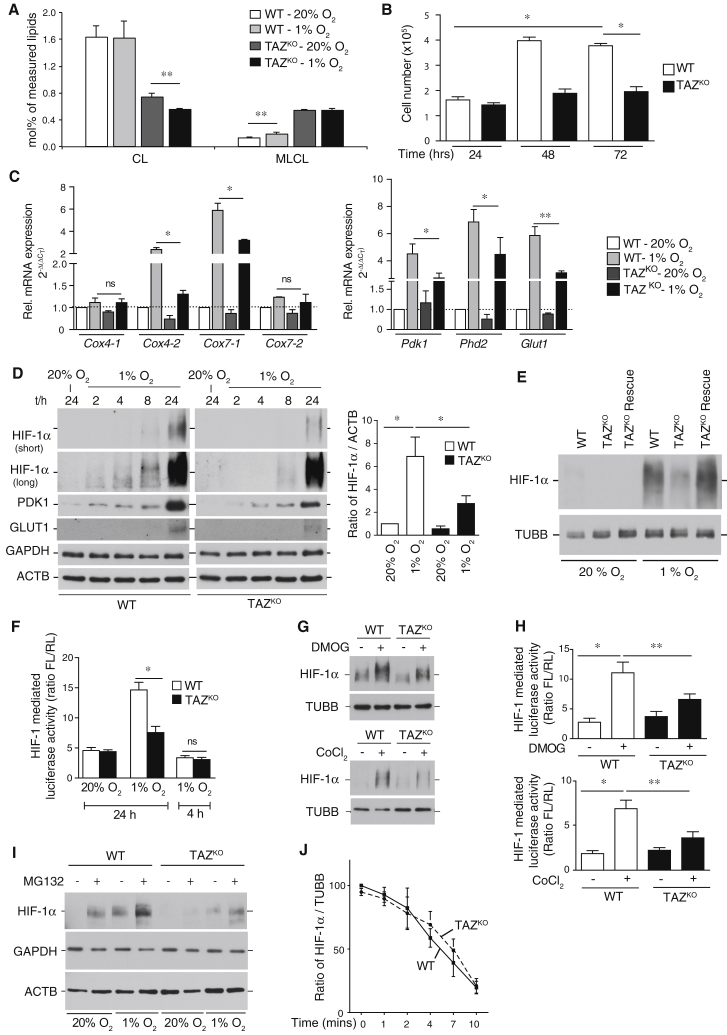
Deficient Cardiolipin Remodeling Causes Blunted HIF-1α Response to Hypoxia (A) Total amounts of cardiolipin and monolysocardiolipin in wild-type (WT) and TAZ^KO^ MEF cells analyzed by mass spectrometry. Means ± SEMs, n = 4 per genotype. ^∗∗^p < 0.01. (B) Cell count of WT and TAZ^KO^ MEF cells cultivated in galactose-containing media. Means ± SEMs, n = 4, ^∗^p < 0.0001. (C) Analysis of gene expression of cytochrome *c* oxidase subunits (left) and canonical HIF-1α target genes (right) in total mRNA isolated from WT and TAZ^KO^ MEF cells subjected to normoxia and hypoxia for 24 hr by qPCR. Means ± SEMs, n = 3, ^∗^p < 0.0294, ^∗∗^p < 0.0001. ns, non-significant. (D) Analysis of steady-state protein levels in whole-cell extracts of WT and TAZ^KO^ MEF cells after exposing to normoxia or hypoxia for the indicated time points by western blotting with antibodies against indicated proteins (left). Quantification of a signal ratio of HIF-1α/β-actin after 24 hr of hypoxia with WT normoxia set to 1 (right). Means ± SEMs, n = 3, ^∗^p < 0.04. (E) Analysis of HIF-1α steady-state levels under hypoxia and normoxia in WT, TAZ^KO^, and TAZ^KO^ Rescue MEF cells. Whole-cell lysates were analyzed by western blotting using antibodies against HIF-1α and β-tubulin. (F) HIF-1α activity measurement using luciferase reporter assay, as described in the STAR Methods for indicated time points under normoxia or hypoxia. Means ± SEMs, n = 3, ^∗^p < 0.0001. ns, non-significant. (G) Steady-state protein levels analysis of whole-cell lysate from WT and TAZ^KO^ MEF cells after treatment with 2 mM DMOG or 50 μM CoCl_2_ for 24 hr by western blotting with antibodies against indicated proteins. n = 3. (H) Luciferase-reporter activity under control of an HIF-1α promoter as described in the STAR Methods after administration of 2 mM DMOG or 50 μM CoCl_2_ for 24 hr. Means ± SEMs, n = 3, ^∗^p < 0.0005, ^∗∗^p < 0.005. (I) Western blot analysis of HIF-1αsteady-state levels in cells treated with 25 μM of proteasomal inhibitor MG132 under normoxia and hypoxia for 8 hr. Antibodies against GAPDH and ACTB were used as a control. (J) Determination of HIF-1α degradation kinetics in WT and TAZ^KO^ MEF cells using cycloheximide chase for the indicated time points after 24 hr of hypoxia exposure. Quantitation of HIF-1α protein levels compared to β-tubulin in WT and TAZ^KO^ MEF cells. Means ± SEMs, n = 3. See also Figures S1 and S2.

To investigate the consequences of a loss of tafazzin on mitochondrial and cellular functions, we used CRISPR/Cas9-mediated gene editing to disrupt the TAZ gene in mouse embryonic fibroblasts (MEFs). The correct targeting of the TAZ gene and the mutations that were caused were confirmed by sequencing of the genomic locus (Figure S1A). Because reliable antibodies against mouse tafazzin are not available, we confirmed loss of tafazzin function in TAZ^KO^ cells by mass spectrometry to demonstrate that these cells displayed reduced cardiolipin levels and the concomitant increase in monolysocardiolipin (MLCL) (Figure 1A) characteristic of TAZ deficiency (Houtkooper et al., 2009). Consistent with a shift in the mitochondrial cardiolipin pool, isolated TAZ^KO^ mitochondria showed a destabilization of respiratory chain supercomplexes and a reduction of the mitochondrial membrane potential (Figures S1C–S1E), as observed before in diverse Barth syndrome models (Brandner et al., 2005, Dudek et al., 2013, McKenzie et al., 2006, Pfeiffer et al., 2003). On glucose-containing medium, TAZ^KO^ cells displayed a mild growth defect that was exacerbated on respiratory medium (Figures 1B and S1F). The enzymatic activity of the respiratory chain complexes is regulated by specific subunits, which allow for adaptation to environmental changes. We found that upon shift of TAZ^KO^ cells to hypoxia (1% O_2_) for 24 hr, the expression of the tested hypoxia-specific isoforms of complex IV subunits (COX4-2, COX7-1) of the respiratory chain was drastically reduced (Figure 1C). The expression of these genes is under control of the hypoxia-inducible transcription factor HIF1 (Fukuda et al., 2007, Hwang et al., 2015). To this end, we assessed expression of established target genes of HIF-1α, including phosphoinositide-dependent protein kinase-1 (Pdk1), Phd2, and glucose transporter type 1 (Glut1), and found that their expression was similarly dampened in the absence of TAZ (Figure 1C). In agreement with these findings, the amount of the HIF-1α protein was significantly reduced in TAZ^KO^ cells compared to wild-type, in which a maximal amount of HIF-1α was apparent after 24 hr of hypoxia (Figure 1D). The phenotype of reduced HIF-1α level in hypoxia could be rescued in TAZ^KO^ Rescue cells, in which the mutations in the TAZ^KO^ were corrected through a second CRISPR-mediated homology repair (HR) event (Figures 1E and S1B). The TAZ^KO^ cells displayed decreased expression of a luciferase-reporter gene under the control of an HIF-1-activated promoter, supporting the functional relevance of the observed reduced HIF-1α levels (Figure 1F).

HIF-1α is a central regulator of a global metabolic transcription program and is therefore tightly regulated at the posttranslational level in the presence of molecular oxygen. HIF-1α is a short-lived protein, which is hydroxylated by PHDs and subsequently degraded through the ubiquitin/proteasome pathway. Accordingly, PHD inhibitors (dimethyloxaloylglycine [DMOG] and CoCl_2_) cause stabilization of HIF-1α even under normoxic conditions (Jaakkola et al., 2001, Piret et al., 2002). To address whether the reduced levels of HIF-1α in the absence of tafazzin were due to increased turnover, we treated TAZ^KO^ cells with PHD inhibitors CoCl_2_ and DMOG and the proteasome inhibitor MG132. While these treatments robustly increased the levels of HIF-1α in wild-type (WT) cells, HIF-1α levels were significantly lower in TAZ^KO^ cells (Figures 1G and 1I). Similarly, transcriptional activation, measured by HIF-1-driven luciferase activity levels, did not recover to WT levels in CoCl_2_- and DMOG-treated TAZ^KO^ cells (Figure 1H). Accordingly, the kinetics of HIF-1α turnover were similar in TAZ^KO^ and WT cells (Figures 1J and S1G). Thus, the decreased HIF-1α levels in TAZ^KO^ cells were not caused by a failure to increase protein stability under hypoxia.

### Cellular Gene Expression Is Altered in the Absence of Tafazzin

**Figure 2:**
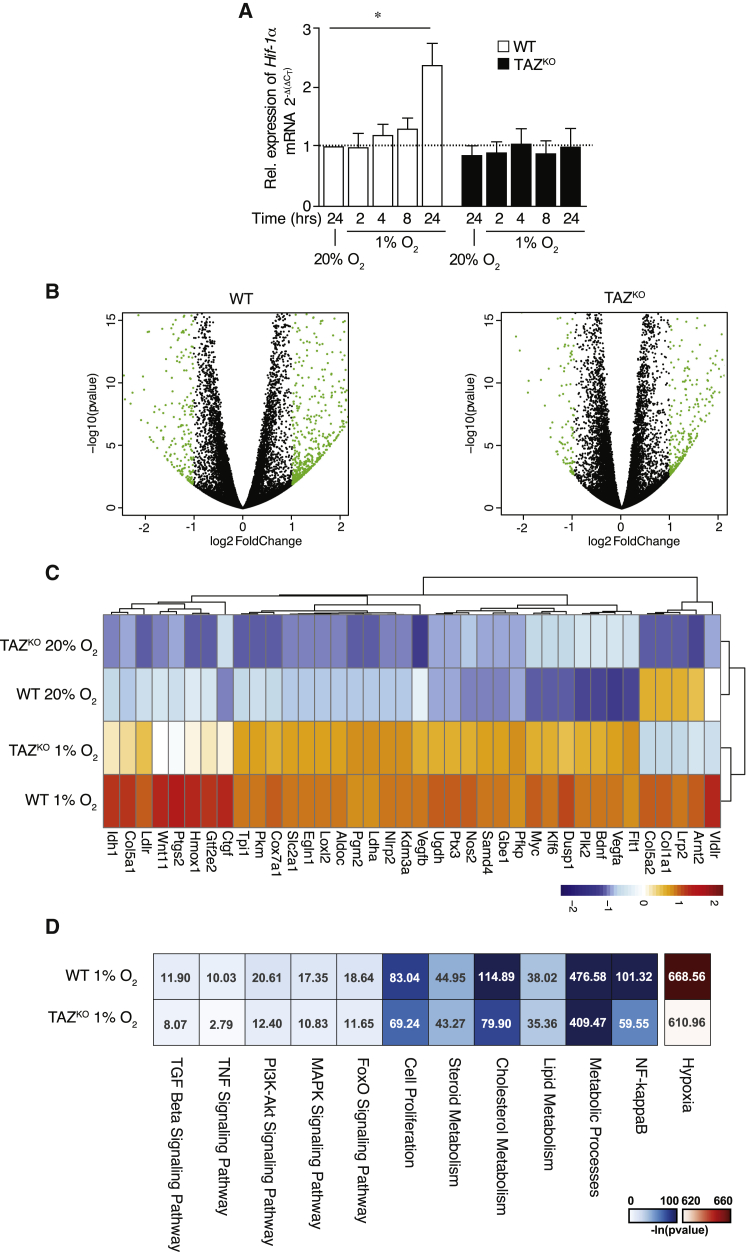
Differential Gene Expression Profiling Using RNA-Seq Reveals Depressed HIF-1α Transcriptional Regulation in TAZ^KO^ Cells (A) qPCR analysis of HIF-1α gene expression from total mRNA isolated at indicated time points from WT and TAZ^KO^ MEF cells under hypoxia and normoxia. Means ± SEMs, n = 3. (B) The volcano plot of log2 fold change versus −log10 p values shows induction of genes upon hypoxia in TAZ^KO^ MEF cells (right) in comparison with WT (left). Genes marked in green are statistically significant, with adjusted p ≤ 0.05 and log2 fold change of 1.0. (C) A heatmap showing the gene expression profile of selected HIF-1α target genes. The samples (mean of all replicates in each group) and genes are clustered with hierarchical clustering using Pearson correlation with average linkage. The expression levels are normalized using variance-stabilizing transformation and then centered and scaled row-wise indicated by the color scale from blue to red (representing low expression to high expression). (D) Heatmap showing the most significant pathways that are enriched in TAZ^KO^ cells compared to WT cells under hypoxia. The color bar represents −log10_e_ p values of the pathways, where darker color represents more significant pathways. See also Tables S1 and S2.

Because the reduced levels of HIF-1α in the absence of TAZ were obviously not linked to the established posttranslational regulatory process, we addressed whether a loss of TAZ affected the transcription of HIF-1α. For this, we analyzed HIF-1α mRNA levels in WT and TAZ^KO^ cells under normoxia and hypoxia. These qPCR analyses showed that in WT cells, expression of HIF-1α increased during hypoxia kinetically. However, this increase was not observed in the absence of TAZ, and mRNA levels remained at a basal expression rate (Figure 2A).

Using an unbiased alternative approach, we performed RNA sequencing (RNA-seq) analyses from WT and TAZ^KO^ cells grown under hypoxia and normoxia. In WT cells, the gene expression profile changed significantly upon the shift to oxygen-limiting conditions. This response was drastically dampened in TAZ^KO^ cells (Figure 2B; Tables S1 and S2). A strong reduction in hypoxia response was observed in particular for HIF-1α (Tables S1 and S2) and its target genes (Figure 2C). Based on these data, we analyzed subunit swapping of normoxia-specific respiratory chain subunits (RCF1B and COX4-1) to hypoxia-specific isoforms (RCF1A and COX4-2). In agreement with the transcriptome data, the protein amounts of hypoxia-specific subunits were reduced in TAZ^KO^ (Figure S2A). The TAZ-dependent changes in expression pattern extended beyond HIF-1α−induced genes. Several signaling and metabolic pathways, including nuclear factor κB (NF-κB) pathway targets, were significantly dampened in the absence of tafazzin (Figure 2D). The transcriptome data also revealed a selective reduction in TAZ expression in WT cells under hypoxic conditions, while other enzymes involved in cardiolipin biosynthesis were not decreased (Table S1). We confirmed this finding by qPCR (Figure S2B). In agreement with this, lipidome analyses of WT cells revealed an increased amount of MLCL under hypoxia (Figures 1A and S2C). A hypoxia-dependent increase in MLCL levels was not observed in TAZ^KO^ cells; however, cardiolipin was significantly reduced under these conditions. Likewise, lipid profiles of WT and TAZ^KO^ cells under hypoxic conditions were significantly changed (Figures S2C–S2E). Moreover, ceramide levels, which have been implicated in mitochondrial stress response signaling (Kim et al., 2016), increased in hypoxia but remained similar between WT and TAZ^KO^ (Figure S2E). We concluded that loss of tafazzin leads to alterations in gene expression and dampened transcription of HIF-1α. In addition, under conditions of limited oxygen supply, cells appear to increase the level of MLCL by reduced expression of TAZ.

### NF-κB Activation Is Compromised when Cardiolipin Remodeling Is Affected

**Figure 3:**
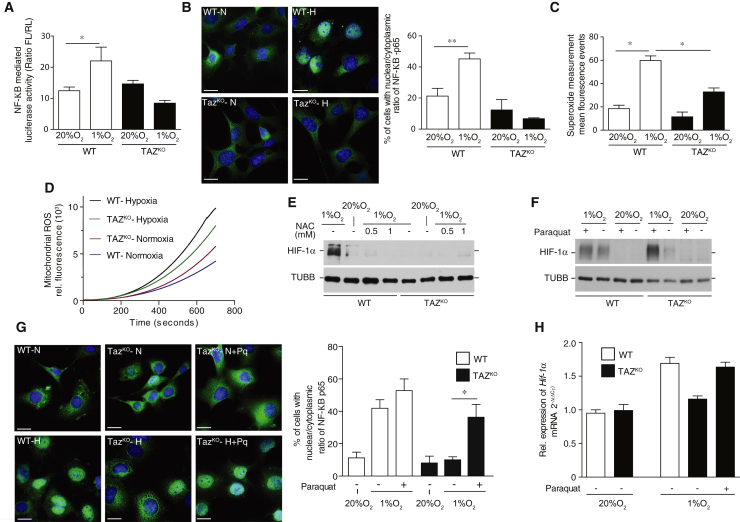
NF-κB-Mediated Gene Induction of HIF-1α Is Required for Adequate Response to Hypoxia (A) NF-κB activity measurement using luciferase reporter assay as described in the STAR Methods for 24 hr under normoxia or hypoxia. Means ± SEMs, n = 4, ^∗^p < 0.0177; ns, non-significant. (B) Determination of NF-κB nuclear translocation under normoxia and hypoxia for 24 hr in WT and TAZ^KO^ MEF cells by immunostaining the cells with antibodies against NF-κB-p65 (green) and DAPI (blue). Bars, 15 μm. N, normoxia; H, hypoxia. Quantification of cells with nuclear NF-κB to cytoplasmic localization as described in the STAR Methods. Means ± SEMs, n = 3, ^∗∗^p < 0.05. (C) Flow cytometry detection of changes in mitochondrial ROS levels by staining WT and TAZ^KO^ MEF with MitoSOX Red after exposure to hypoxia or normoxia for 24 hr. Means ± SEMs, n = 4, ^∗^p < 0.05. (D) Quantitation of ROS production over time in isolated mitochondria from WT and TAZ^KO^ MEF cells exposed to hypoxia or normoxia for 24 hr using the ROS-specific fluorescence-based sensor 2',7'-dichlorodihydrofluorescein diacetate (H_2_DCFDA). Means ± SEMs, n = 3. (E) Western blot analysis of HIF-1α protein levels under hypoxia in the presence and absence of 0.5 and 1 mM NAC using anti-β-tubulin antibody as a control. (F) Determination of HIF-1α protein levels in comparison to β-tubulin under hypoxia in the presence and absence of 450 μM paraquat in WT and TAZ^KO^ MEFs. (G) Analysis of NF-κB nuclear translocation in WT and TAZ^KO^ MEFs under hypoxia and normoxia in the presence and absence of 450 μM paraquat. NF-κB was detected by immunostaining with antibodies against NF-κB -p65 (green) and DAPI (blue). Bars, 15 μm. N, normoxia; H, hypoxia. The graph on the right shows quantification of cells with NF-κB to cytoplasmic localization that has been done as described in the STAR Methods. Means ± SEMs, n = 3, ^∗^p < 0.05. (H) qPCR analysis of HIF-1α gene expression from total mRNA isolated from WT and TAZ^KO^ MEF cells under hypoxia and normoxia in the presence and absence of 450 μM paraquat. Means ± SEMs, n = 3. See also Figure S3.

The expression of HIF-1α is under control of the transcription factor NF-κB (van Uden et al., 2008). The RNA-seq data indicated drastic changes in NF-κB-dependent gene expression (Figure 2D). To this end, we assessed whether NF-κB signaling was compromised in TAZ^KO^ cells and analyzed NF-κB activity with a reporter gene assay. While luciferase under control of a promoter with an NF-κB-binding element was >2-fold induced in WT cells in hypoxia, this activation did not occur in TAZ^KO^ cells (Figure 3A). Because NF-κB translocates from the cytoplasm to the nucleus upon activation, we monitored the cellular distribution of NF-κB in normoxia and hypoxia. NF-κB efficiently and specifically accumulated in the nucleus under hypoxic conditions in WT cells. In contrast, in TAZ^KO^ cells nuclear translocation did not occur, which is indicative of inefficient NF-κB activation (Figure 3B).

Reactive oxygen species (ROS) are among the key stimuli that activate NF-κB at the plasma membrane in T cells and epithelial cells (Schreck et al., 1991). Therefore, we assessed superoxide anion production in MEF cells in normoxia and hypoxia. First, we measured superoxide anion production by flow cytometry in WT and TAZ^KO^ cells. In WT cells, the production of superoxide anion was drastically increased in hypoxia. In contrast, this hypoxia-specific increase in superoxide levels was diminished in TAZ^KO^ cells by approximately 50% (Figure 3C). Second, we measured the kinetics of ROS production in purified mitochondria from WT and TAZ^KO^ cells grown in hypoxia or normoxia. Mitochondria from normoxic TAZ^KO^ cells displayed increased ROS production compared to WT cells. The production of ROS in WT mitochondria increased drastically when cells were grown under hypoxic conditions. However, the production of mitochondrial ROS increased significantly less in the TAZ^KO^ sample than in the WT (Figure 3D). The reduction in ROS emission in TAZ^KO^ mitochondria is in line with a reduction in the mitochondrial inner membrane potential (Figure S3A). In summary, in hypoxia TAZ deficiency leads to decreased mitochondrial ROS production compared to WT.

To assess the role of ROS signals in the induction of HIF-1α, we treated cells with ROS-quenching *N*-acetyl cysteine (NAC). NAC treatment blocked HIF-1α activation under hypoxic growth conditions (Figure 3E). Similar results were obtained using the mitochondria-targeted ROS scavenger MitoTEMPO (Figure S3B). We reasoned that if defective NF-κB activation in TAZ^KO^ cells was linked to reduced ROS production in mitochondria, then we should be able to complement HIF-1α expression by increasing mitochondrial ROS production chemically. To this end, TAZ^KO^ cells were treated with paraquat to increase ROS production by the respiratory chain (Figures S3C and S3D). The response of TAZ^KO^ to paraquat appeared quantitatively less than in WT MEF cells. This is likely due to alterations in the respiratory complexes upon cardiolipin deficiency (Figure S1C). In the paraquat-treated cells, HIF-1α reached WT levels under hypoxic conditions (Figure 3F). Moreover, paraquat treatment also complemented the nuclear translocation phenotype of NF-κB in TAZ^KO^ cells (Figure 3G) and the decrease in HIF-1α mRNA levels in TAZ^KO^ cells (Figure 3H). In addition, paraqaut treatment increased the activity of NF-κB in TAZ^KO^ cells upon hypoxia (Figure S3E). Similarly, antimycin A and rotenone treatment to induce ROS through complex III and complex I corrected NF-κB nuclear translocation in TAZ^KO^ cells (Figure S4A). These analyses demonstrated that mitochondrial ROS production needs to reach a critical threshold to activate NF-κB signaling in hypoxia and thus to trigger a physiologically adequate response of HIF-1α.

### Blunted HIF-1α Signaling Causes Increased Susceptibility to Heart Failure in Barth Syndrome Mouse Model

**Figure 4:**
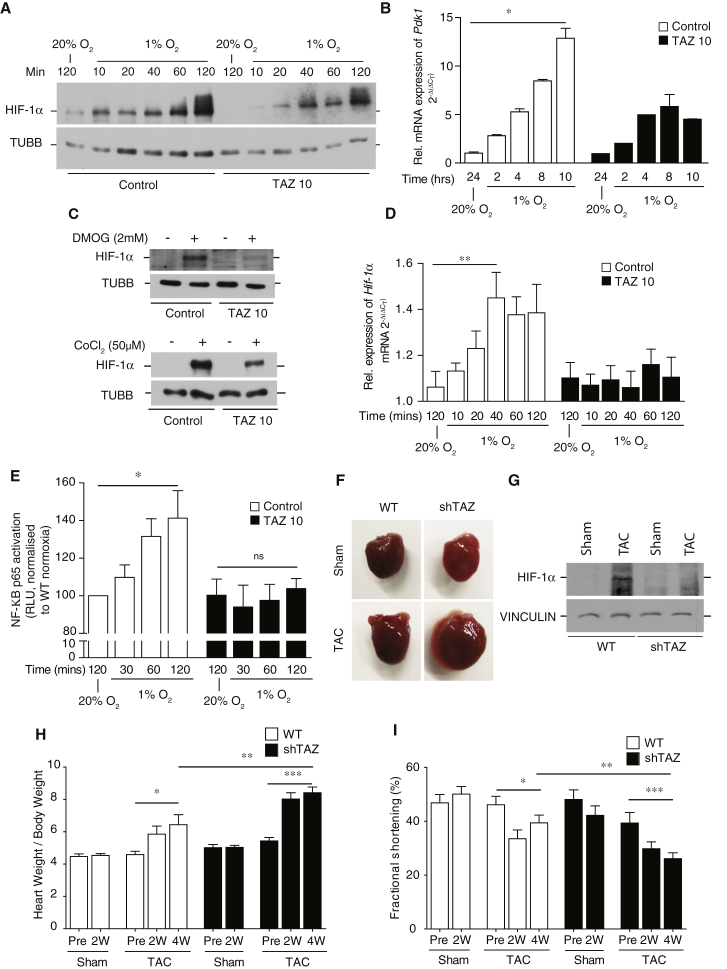
Decreased HIF-1α Signaling in Barth Syndrome Patient iPS-CM and shTAZ Mice (A) Analysis of HIF-1α protein stability in control versus TAZ 10 iPS-CM after exposure to normoxia or hypoxia for the indicated time points. (B) qPCR analysis of the HIF-1α target *Pdk1* in total mRNA isolated from control versus TAZ 10 iPS-CM subjected to normoxia and hypoxia for 8 hr by qPCR. Means ± SEMs, n = 3, ^∗^p < 0.01. (C) Determination of HIF-1α protein stability in whole-cell lysates from control versus TAZ 10 iPS-CM upon administration of 2 mM DMOG or 50 μM CoCl_2_ for 24 hr by SDS-PAGE followed by western blotting with antibodies against HIF-1α and β-tubulin. n = 3. (D) qPCR analysis of HIF-1α gene expression from total mRNA isolated at indicated time points from control versus TAZ 10 iPS-CM under hypoxia and normoxia conditions. Means ± SEMs, n = 3, ^∗∗^p < 0.03. (E) Quantification of NF-κB-p65 activation in nuclear extracts isolated from control and TAZ 10 iPS-CM after hypoxia and normoxia for the indicated time points using ELISA-based NF-κB p65 transcription factor assay kit. Means ± SEMs, n = 3, ^∗^p < 0.05; ns, non-significant. (F) Representative picture of mouse hearts dissected from WT and shTAZ after 4 weeks of sham and TAC surgery. (G) Determination of HIF-1α protein levels in tissue lysates from hearts of WT and shTAZ mice after 4 weeks of sham and TAC surgery. (H) Quantification of the heart weight-to-body weight ratio in WT and shTAZ before (pre) and after 2 weeks (2W) or 4 weeks (4W) of sham and TAC surgery. Means ± SEMs, n = 8 in WT, n = 12 in shTAZ, ^∗∗^ unpaired t test: p < 0.05, ^∗^ and ^∗∗∗^ paired t tests: p < 0.05. (I) Quantification of the percentage of fractional area shortening in WT and shTAZ before (pre) and after 2 weeks (2W) or 4 weeks (4W) of sham and TAC surgery. Means ± SEMs, n = 8 in WT, n = 12 in shTAZ, ^∗∗^ unpaired t test: p < 0.05, ^∗^ and ^∗∗∗^ paired t tests: p < 0.05. See also Figure S3.

To this end, we addressed whether HIF-1 signaling was similarly affected in disease models of Barth syndrome using patient induced pluripotent stem cell (iPSC)-derived cardiomyocytes (TAZ 10) (Dudek et al., 2016). HIF-1α stabilization displayed significantly faster kinetics in these cells compared to MEF cells. Moreover, in TAZ 10 cardiomyocytes, a significant reduction in HIF-1α protein level was apparent under hypoxic conditions, which went along with a blunted induction of its target genes (Figures 4A and 4B). As expected, inhibitors of HIF-1α hydroxylation did not rescue HIF-1α protein levels, excluding that increased HIF-1α turnover was the cause for reduced protein levels in Barth syndrome (BTHS) cardiomyocytes (Figure 4C). In agreement with this, HIF-1α had a 7-min half-life in BTHS and control cardiomyocytes. However, under hypoxia, HIF-1α gene induction was affected in TAZ 10 cardiomyocytes (Figure 4D). Reporter gene assays of NF-κB activity in TAZ 10 cardiomyocytes revealed decreased transcriptional reporter activation (Figure 4E). Accordingly, also in patient-derived cardiomyocytes, HIF-1α gene induction in hypoxia requires the activation of NF-κB signaling.

HIF-1α plays key roles in signaling pathways in the heart muscle in response to stress (Bishop and Ratcliffe, 2015). We therefore analyzed the response of cardiac tissue to pressure overload induced by transversal aortic constriction (TAC) in the BTHS mouse model (shTAZ). Here, we found a drastic morphological difference between control and BTHS mice in response to pressure overload (Figure 4F). While HIF-1α levels increased in control mouse hearts, this effect was severely reduced in shTAZ mice (Figure 4G). The heart responds to TAC surgery with hypertrophy in an effort to compensate the increased afterload. Long-term exposure to mechanical stress results in decompensation and heart failure. Failing HIF-1α signaling in shTAZ mouse hearts causes significant progression into decompensated heart failure upon TAC surgery. The hearts of shTAZ mice show a significant augmented hypertrophy response (Figures 4H, S4B, and S4C), while cardiac function, as determined by fractional shortening and ejection fraction, strongly decreased (Figures 4I and S4D). Moreover, the hearts of shTAZ mice displayed an increase in *Bnp* (brain natriuretic peptide) expression, which is indicative of transition to heart failure (Figure S4E). These data demonstrate that in the absence of tafazzin, mice hearts lost the capacity to adapt to pressure overload challenges and that HIF-1α function is compromised under these conditions. However, considering the broad impact of loss of tafazzin on metabolism and signaling processes, the observed reduced adaptive capacity of the heart may be influenced by other processes in addition to the reduction in HIF-1α functionality.

### Conclusions

Our results reveal a link between mitochondrial cardiolipin remodeling and HIF-1 signaling. Previous work suggested a connection between mitochondria-derived ROS and HIF-1α stabilization. However, the suggested mechanistic concept predicted an effect of ROS on PHD enzymatic function and thus conveyed a posttranslational effect on HIF-1α (Chandel, 2010, Chandel et al., 2000). Here, we demonstrate that ROS in fact trigger a transcriptional response that is communicated from mitochondria to the nucleus through NF-κB. Such a mechanism of ROS-induced activation of NF-κB phenocopies the activation cascade for NF-κB in inflammatory responses and tumor tissue (Schreck et al., 1991, Taylor and Colgan, 2017). While our observations provide principal mechanistic insight into how mitochondrial retrograde signaling in hypoxia occurs, we also demonstrate that this pathway is physiologically relevant. In BTHS models, HIF-1α activation is compromised because mitochondrial ROS levels drop beyond the activation threshold for NF-κB. At the same time, we recognize regulatory feedback under conditions of hypoxic growth at which TAZ expression is downregulated to increase MLCL in mitochondria. These findings provide a mechanistic framework for mitochondrial retrograde signaling and demonstrate that in BTHS signaling defects affect the ability of the heart muscle to cope with functional challenges.

## STAR★Methods

### Key Resources Table

**Table:** 

REAGENT or RESOURCE	SOURCE	IDENTIFIER
**Antibodies**		

Mouse anti-HIF-1α	Novus Biologicals	Cat#NB100-479; RRID: AB_10000633
Human anti-HIF-1α	BD Biosciences	Cat#610959; RRID: AB_398272
anti-PDK1	Stressgen Biotechnologies	Cat#KAPPK112; RRID: AB_2039453
anti-GLUT1	Novus Biologicals	Cat#NB300-666; RRID: AB_10000485
anti-PHD3	Novus Biologicals	Cat#NB100-303; RRID: AB_10003302
anti-GAPDH	Abcam	Cat#ab75834; RRID: AB_1310254
Anti-β-actin (ACTB)	Sigma	Cat#A5441; RRID: AB_476744
anti-β-tubulin (TUBB)	Abcam	Cat#ab6064
Anti-COX4-2	Proteintech	Cat#11463-1-AP; RRID: AB_2085287
anti-COX4-1	Selfmade	PR-1522
anti-RCF1A	Selfmade	PR-3976
anti-RCF1B	Selfmade	PR-3977
anti-TIM23	Selfmade	PR-1526
anti-ATP5B	Selfmade	PR-4826
anti-VDAC3	Selfmade	PR-1514
anti-Tom70	Selfmade	PR-3280
anti-MnSOD	Novus Biologicals	Cat#NB100-1992; RRID: AB_535862
Anti-NF-kappaB p65	Cell Signaling	Cat#8242S; RRID: AB_10859369
anti-Tafazzin	Abcam	Cat#ab93362; RRID: AB_10562289
Secondary antibody Goat anti-rabbit IgG-488 Alexa fluor	Thermo scientific	Cat#A-11034; RRID: AB_2576217
Goat anti-mouse HRP-coupled secondary antibody	Jackson ImmunoResearch	Cat#115-035-146; RRID: AB_2307392
Goat anti-rabbit HRP-coupled secondary antibody	Jackson ImmunoResearch	Cat#115-035-144

**Chemicals, Peptides, and Recombinant Proteins**		

GeneJuice	Merck	Cat#70967-3
Lipofectamine 2000	ThermoFisher Scientific	Cat#11668019
Trizol	ThermoFisher Scientific	Cat#15596026
DAPI Fluoroshield	Sigma-Aldrich	Cat#F6057
CM-H_2_DCFDA	ThermoFisher	Cat#C6827
MitoSOX Red	ThermoFisher	Cat#M36008
TMRM	ThermoFisher	Cat#T668
Paraquat	Sigma	Cat#36541
DMOG	ENZO	Cat#BML-EI 247-0050
CoCl_2_	Fluka	Cat#60820
N-acetylcysteine (NAC)	Sigma	Cat#A7250
Antimycine A	Sigma	Cat#A8674
Rotenone	Sigma	Cat#R8875
MG132	Cayman via BertiPharma	Cat#10012628
Mitotempo	Sigma	Cat#C1988
DAPI Fluoroshield	Sigma-Aldrich	Cat#F6057
CM-H_2_DCFDA	ThermoFisher	Cat#C6827

**Critical Commercial Assays**		

QuikChange Site-Directed Mutagenesis Kit	Agilent	Cat#210515
KOD Hot Start DNA Polymerase	Merck	Cat#71086-3
First Strand cDNA Synthesis kit	ThermoFisher Scientific	Cat#K1612
SensiMix SYBR Low-Rox One Step kit	Bioline	Cat#QT625-05
Dual-luciferase reporter assay system	Promega	Cat#E1910
Nuclear Extraction Kit	Abcam	Cat# ab113474
NF-κB p65 transcription factor assay kit	Abcam	Cat# ab207221

**Deposited Data**		

RNA sequencing data	Gene Expression Omnibus	Acc# GSE119775

**Experimental Models: Cell Lines**		

Mouse: MEF cells	ATCC	N/A
Mouse: MEF cells TAZ^KO^	This paper	N/A
Mouse: MEF cells TAZ^KO^ Rescue	This paper	N/A
Human: iPSC WTD2.3	Dudek et al., 2016	N/A
Human: iPSC TAZ 10.3	Dudek et al., 2016	N/A

**Experimental Models: Organisms/Strains**		

Mouse: ROSA26H1/tetO-shRNA:TAZ	Acehan et al., 2011	MGI:4887237

**Oligonucleotides**		

gRNA1 sequence: TAGGCCCATGACGACGCTGC	This paper	N/A
gRNA2 sequence: TGTGAAGTGGCCATTCCCCG	This paper	N/A
ssODN Repair template: GGCGGGGCCGGAGCCGGAGGAGAT GCCCCTCCATGTGAAGTGGCCCTTTCCTGCAGTTCCTAGACT CACCTGGACTCTAGCCAGCAGCGTCGTCATGGGCCTAGTTGG CACCTACAGCTGCTTCTGGACCAGTGAGTGGCAAAAGGCCAA	This paper	N/A

**Recombinant DNA**		

Plasmid: pH3SVL HIF-dependent Firefly luciferase reporter	Wanner et al., 2000	N/A
Plasmid: pRLSV40 Renilla luciferase reporter	Promega	Cat#E2231
Plasmid: pNF-κB-Luc	Clontech	Cat#631743

**Software and Algorithms**		

ImageJ 1.47v	NIH	RRID: SCR_003070
Geneious	Biomatters Ltd	RRID: SCR_010519
Prism5	GraphPad Software	RRID: SCR_015807
FastQC	Babraham Bioinformatics	RRID: SCR_014583
rna-STAR (version STAR_2.5.2b)	Dobin et al., 2013	RRID: SCR_004463
HTSeq	Anders et al., 2015	RRID: SCR_005514
RUVSeq	Risso et al., 2014	RRID: SCR_006263
DESeq2	Love et al., 2014	RRID: SCR_015687

### Contact for Reagent and Resource Sharing

Further information and requests for reagents may be directed to, and will be fulfilled by the corresponding author Peter Rehling (Peter.Rehling@medizin.uni-goettingen.de).

### Experimental model and subject details

#### Mice

Maintenance of all mice and the study on them were performed according to the guidelines from the German Animal Welfare Act and approved by the Landesamt für Verbraucherschutz und Lebensmittelsicherheit, Niedersachsen, Germany (AZ: 33.9-42502-04-15/1991). Administration of doxycycline (625 mg/kg) was done as part of the standard rodent chow to WT C57BL/6J mice and transgenic (ROSA26H1/tetO-shRNA:TAZ) animals (Acehan et al., 2011, Soustek et al., 2011). Female mice were treated with doxycycline 1 week before mating and doxycycline was withdrawn during mating to avoid male infertility. Once copulatory plugs were detected Doxycycline treatment was resumed and the pups were continuously treated until 2 months of age. The genotype of the pups was assessed by PCR, as described previously (Acehan et al., 2011). For Transverse aortic constriction (TAC) female mice of an age between 8 and 12 weeks were used for experiments. TAC was performed by surgical placement of a suture around the transverse aorta sized to a 26G needle, as described previously (Gröschel et al., 2017). Mice were subjected either to TAC or a sham operation. The appropriate painkillers were administered subcutaneously pre and post surgeries. Mice were euthanized at 2 or 4 weeks after surgery for tissue harvest and analysis. The echocardiography was performed prior to surgery as well at 2 weeks or 4 weeks after intervention prior to sacrificing the animals. The experiments were not randomized. The investigators were not blinded to allocation during experiments and outcome assessment. For transthoracic echocardiography, Vevo2100 (VisualSonics, Toronto, Canada) system with a 30-MHz center frequency transducer was used. In short, animals were anesthetized with 3% isoflurane and temperature-, respiration, and ECG controlled anesthesia was maintained with 1.5% isoflurane. Two dimensional cine loops with frame rates of > 200 frames/s of a long-axis view and a short-axis view at mid-level of the papillary muscles as well as M-mode loops of the short-axis view were recorded. Thicknesses of the septum, the posterior myocardial wall, the inner diameter of the left ventricle (LVED), and the area of the left ventricular cavity (area) were measured in systole (s) and diastole (d) from the short-axis view according to standard procedures (Collins et al., 2003). Maximal left ventricular length (L) was measured from the long-axis view. Systolic and diastolic left ventricular volumes were calculated using the area**–**length method, and the ejection fraction (EF) was calculated out of the volumes. Measurements were obtained by an examiner blinded to the genotype of the animals.

#### Cell lines and cultivation

SV40-large T immortalized and H-ras transformed MEF cells were cultured in DMEM, supplemented with 10% [v/v] fetal bovine serum (FBS) (GIBCO, Invitrogen) at 37°C under a 5% CO_2_ humidified atmosphere. Lipofectamine 2000 (Thermo Scientific) was used for transfections, which were performed according to manufacturer’s recommendations. Briefly, approximately 300,000 cells/25 cm^2^ were transfected using 4 μL of transfection reagent and 1 μg of DNA TAZ^KO^ cell line was generated using the CRISPR/Cas9 technology as previously described (Ran et al., 2013). Briefly, oligonucleotides 5′-TAGGCCCATGACGACGCTGC-3′ and 5′-GCAGCGTCGTCATGGGCCTA-3′ containing the guide sequences were annealed and ligated into the pX330 vector. WT MEFs cells were co-transfected with pX330 and with the pEGFP-N1 plasmid. After three days, single cells expressing GFP were sorted by flow cytometry into 96 well plates. After colony expansion single colonies were screened by sequencing. TAZ^KO^ Rescue was generated through CRISPR/Cas9 mediated repair of the mutant alleles. The guide RNA sequence used for this purpose is 5′-TGTGAAGTGGCCATTCCCCG-3′. The guides were cloned into pX330-GFP (GFP expression through an independent CMV promoter). TAZ^KO^ cells were co-transfected with pX330-GFP and ssODN repair template, 5′-GGCGGGGCCGGAGCCGGAGGAGATGCCCCTCCATGTGAAGTGGCCCTTTCCTGCAGTTCCTAGACTCACCTGGACTCTAGCCAGCAGCGTCGTCATGGGCCTAGTTGGCACCTACAGCTGCTTCTGGACCAGTGAGTGGCAAAAGGCCAA-3′. The cells were screened as mentioned above for the correction.

For studying cell proliferation under different growth conditions, 1x10^5^ WT and TAZ^KO^ MEF cells were initially plated in media containing either glucose (9 g/L) or galactose (0.9 g/L). The cells were counted after every 24 hours using a haemocytometer.

#### Control and patient-specific iPS-CM

Generation of the patient-specific iPS-CM (control and TAZ 10) was done as described previously (Dudek et al., 2016, Lian et al., 2013). The control iPS-CM line was derived from a healthy individual, whereas TAZ 10 was generated from a BTHS patient carrying the mutation c.590 G > T in the TAZ gene (Dudek et al., 2013). All hiPSC lines were expanded as adherent cultures in feeder- dependent cultures on Geltrex-coated cell culture dishes in the presence of chemically defined E8 Essential Medium, (Life Technologies). Differentiation of hiPSC to cardiomyocytes was performed over a period of 30 days, based on small molecules-mediated canonical Wnt pathway modulation as reported previously. Enrichment for cardiomyocytes was performed by adding 4 mM lactate in substitution of glucose for 6 days between days 20 and 30 (Tohyama et al., 2013). Cultures of iPS-CMs were then enzymatically dissociated into single cells using 0.25% trypin-EDTA (Life technologies) and further plating and culturing CMs was done in RPMI 1640 (HEPES/GlutaMax) (Life technologies) medium supplemented with B27 and insulin (Life technologies).

### Method Details

#### Cell compound treatments

For inducing hypoxia, the MEF cells or iPS-CM were incubated under defined hypoxic conditions (1% O_2_), an *in vivo* hypoxia workstation (Baker Ruskinn, The Baker Company, Bridgend, South Wales, United Kingdom). For normoxia, the cells were maintained in a regular cell culture incubator (with 20% O_2_). For inhibiting PHD activity under normoxic condition the cells were treated either with 50 μM CoCl_2_ or 2 mM DMOG for 24 hours. MEF cells were treated with 450 μM Paraquat, 20 hours after hypoxia induction. The treatment was continued for additional 4 hours under hypoxic condition. Other oxidants such as Antimycin A and Rotenone were used at 0.2 mM each for similar durations. The cells were then processed for cell lysate preparation or immunostaining. For quenching ROS levels, N-acetylcysteine (NAC) (1 M prepared in culture medium, pH adjusted to 7.2) was added to culture medium at a final concentration of 0.5 or 1 mM for 24 hours during hypoxia. Similarly, MitoTempo was used at 0.05 mM for 24 hours during hypoxia. To block proteasomal activity of HIF-1α the cells were treated with a final concentration of 25 μM MG132, added to culture medium of the cells for 8 hours under hypoxia or normoxia. For determining the half-life of endogenous HIF-1α, 50 μM of cycloheximide (CHX) was added to the cells inside the hypoxia workstation after 24 hours of hypoxia treatment. The cells were then taken out after 1, 2, 4, 7 and 10 mins of CHX treatment for cell lysate preparation.

#### Luciferase reporter gene assay

For determining HIF-1 activity, cells were co-transfected with 100 ng of the HIF-dependent *Firefly* luciferase reporter gene construct pH3SVL as described previously (Wanner et al., 2000) and 5 ng of the constitutively active pRLSV40 *Renilla* luciferase reporter plasmid (Promega, Madison, WI, USA) to control for differences in transfection efficiency. Alternatively, for NFκB activity analysis the cells were co-transfected with 100 ng of the NFκB-dependent firefly luciferase reporter gene construct, pNF-κB-Luc (Clontech) and 5 ng of pRLSV40 *Renilla* luciferase reporter plasmid (Promega, Madison, WI, USA) to control for differences in transfection efficiency. At 6 hours post-transfection, cells were equally distributed and exposed to 20% or 1% O_2_ for another 24 hours. After washing with PBS and carrying out cell lysis using passive cell lysis buffer (Promega), Firefly and *Renilla* luciferase reporter activities were determined using the dual-luciferase reporter assay system according to the manufacturer’s instructions (Promega) using a microplate luminometer (Berthold, Regensdorf, Switzerland).

#### Immunofluorescence analyses

Immunofluorescence staining was performed using standard protocols (Chowdhury et al., 2014). Cells grown on coverslips were kept in hypoxia or normoxia for 24 hours and were fixed in 4% paraformaldehyde (Roth). Cells were permeabilized with 0.2% Triton X-100 (Roth). After washing with PBS, non-specific binding sites were blocked with 1% BSA + 1% goat serum in PBS and stained with anti-NFκB-p65 rabbit monoclonal antibody (#8242, Cell signaling, 1:400) for one hour. After washing coverslips were further incubated for 2 hours with the secondary antibody Goat anti-rabbit IgG-488 Alexa fluor (A-11034 Thermo scientific). After final washing, cells were mounted in histology mounting medium containing DAPI (Fluoroshield; Sigma-Aldrich, F6057). Fluorescence micrographs were captured using a DeltaVision Spectris fluorescence microscope (Applied Precision) at 60X magnification, equipped with a FITC (excitation 475/28, emission 523/36) and DAPI (excitation 390/18, emission 435/48) filter set. A series of 15-20 sections with 0.5-μm spacing along the Z axis were taken. Images were deconvoluted and projections were created from stacks by merging the individual slices using the softWoRx software (Applied Precision).

The imaging analysis was performed as follows, relying on self-written routines in MATLAB (The Mathworks, Inc., Natick, MA, USA). Nuclei were identified by DAPI staining. Each individual nucleus was identified using an automatic thresholding procedure that removed all signals close to background fluorescence, thereby maintaining only nuclei. The immunostaining intensity within the regions of interest representing the nuclei was then determined automatically, within the respective images. To obtain the fluorescence levels within the cytosol of the respective cells, each region of interest corresponding to a nucleus was dilated using an appropriate scaling factor (which was identical for all cells), thus obtaining a circular region of interest that bordered each nucleus. The signal intensity in this new region of interest was then automatically measured, and was compared to the signal within the respective nucleus.

#### ROS measurement with H_2_DCFDA

CM-H_2_DCFDA (5-(and-6)-chloromethyl-2′,7’-dichlorodihydro fluorescein diacetate, acetyl ester) was used as a detector of ROS as described previously (Dudek et al., 2016). The mitochondria isolated from MEFs were resuspended in 10 μM H_2_DCFDA in assay buffer (20 mM Tris**-**HCl, 150 mM NaCl, 1% Triton X-100, pH 7.4). The changes in fluorescence at an excitation wavelength of 498 nm and an emission wavelength of 525 nm were determined using a fluorescence spectrophotometer (Hitachi F-7000) for 700 s. For each sample of a given experiment, the measurement was performed in triplicate, and expressed as arbitrary fluorescence intensity units (AFU).

#### Flow cytometry analyses

The BD-Canto flow cytometer (Becton Dickinson) was used for flow cytometry. Analyses were performed on 10,000 gated events and numeric data were processed using the FACS-Diva software. For estimation of mitochondrial membrane potential, TMRM was used at a concentration of 100 nM. Cells were stained with MitoSOX Red at a concentration of 3 μM for measuring mitochondrial superoxide anion production. In each case, 10^6^ cells were stained with the respective dyes after the indicated treatments using the vendor`s protocols. For cells treated with hypoxia the entire staining procedure was carried out inside the hypoxia chamber under stable and controlled 1% O_2_ oxygen concentration. After fixation with 4% paraformaldehyde, the final steps were then carried out outside the workstation in normoxic conditions.

#### NF-κB activity assay

A total of 1 × 10^7^ TAZ 10 iPS-CM cells were plated in 10 cm cell culture dishes a day prior to the experiment. The cells were then subjected to hypoxia for the indicated time points. While in hypoxia the cells were harvested using 0.25% Trypsin-EDTA and washed with 1X PBS inside the hypoxia chamber. They were then lysed using the Pre-extraction buffer provided in the Nuclear Extraction Kit (Abcam, Cat. No. ab113474). Post lysis, the following steps were carried outside the hypoxia chamber. The nuclear lysates were then processed for NF-κB p65 transcription factor assay kit (Abcam, Cat. No. ab207221) according to the vendor's protocol. The plate was read in the Centro LB960 Luminometer plate reader (Berthold technologies) and the values were expressed as Relative light units (RLU).

#### Analysis of lipid profiles by mass spectrometry

WT and TAZ^KO^ MEF corresponding to 1.5-2.0 nmol of total lipid were subjected to acidic Bligh and Dyer lipid extractions (Bligh and Dyer, 1959) except for CL and MLCL. Lipid standards were added prior to extractions, using a master mix containing phosphatidylcholine (13:0/13:0, 14:0/14:0, 20:0/20:0; 21:0/21:0, Avanti Polar Lipids), D6-cholesterol (Cambridge Isotope Laboratory), phosphatidylinositol (16:0/16:0, 17:0/20:4, Avanti Polar Lipids), phosphatidylethanolamine, phosphatidylglycerol and phosphatidylserine (each 14:1/14:1, 20:1/20:1, 22:1/22:1, semi-synthesized as described in (Özbalci et al., 2013), sphingomyelin, ceramide and glucosylceramide (d18:1 with N-acylated 15:0, 17:0, 25:0 each, semi-synthesized as described (Özbalci et al., 2013), phosphatidic acid (17:0/20:4, Avanti Polar), and lactosylceramide (d18:1 with N-acylated C12 fatty acid). Plasmalogens were quantified as described in (Gerl et al., 2016). Evaporated lipids were redissolved in 60 μl 10 mM ammonium acetate in methanol and analyzed on a QTRAP6500+ mass spectrometer (Sciex, Canada) with chip-based (HD-D ESI Chip, Advion Biosciences, USA) electrospray infusion and ionization via a Triversa Nanomate (Advion Biosciences, Ithaca, USA) as previously described (Özbalci et al., 2013). Redissolved lipid extracts were diluted 1:10 in 96-well plates (Eppendorf twintec 96, Sigma) prior to measurement. Precursor and neutral loss scanning in positive ion mode was employed to measure glycerophospholipids as described (Özbalci et al., 2013). Remaining samples were subjected to cholesterol determination as described (Özbalci et al., 2013). Data evaluation was done using LipidView (Sciex, Canada) and a software developed in-house (ShinyLipids). For MS analysis of CL and MLCL, lipids were extracted by MTBE extraction as described in (Matyash et al., 2008). Lipid extracts were dried under a gentle nitrogen stream. Dried lipids were re-dissolved in 40% UPLC solvent B (90% 2-propanol/10% acetonitrile /0.1% formic acid/ 10 mM NH_4_HCO_2_) and transferred to silanized glass inserts (Phenomenex) using Hamilton syringes. The glass inserts were placed in Eppendorf tubes and centrifuged in an Eppendorf centrifuge at 9000 rpm for 1.5 minutes. Lipid samples were then subjected to UPLC-ESI-MS/MS analysis performed on an Ultimate^®^ 3000 LC system (Dionex, Thermo Fisher Scientific) coupled to a Q Exactive Hybrid Quadrupole-Orbitrap instrument (Thermo Scientific). For LC separations, a ACQITY UPLC CSH C18 1.7μm, 1.0 × 150 mm column (Waters) was used. The column oven temperature was set to 55°C, the temperature of the autosampler was set to 20°C. The flow rate used was 100 μL/min. The solvent composition used was as follows: 60% acetonitrile/40% H_2_O/0.1% formic acid/10 mM NH_4_HCO_2_ (solvent A), 90% 2-propanol/10% acetonitrile /0.1% formic acid/ 10 mM NH_4_HCO_2_ (solvent B). The starting solvent composition was 40% solvent B/60% solvent A. The conditions of the gradient were as follows: 3 min: 50% solvent B, 9 min: 54% solvent B, 9.1 min: 70% solvent B, 17-22 min: 90% solvent B and 22.5-30 min: 40% solvent B. The MS analyses were performed in the negative ion mode. The following ESI source parameters were used: sheath gas flow rate: 4 (a.u.), auxilliary gas flow rate: 0, sweep gas flow rate: 0, spray voltage: 4 kV, capillary temperature: 320°C, S-lens RF level: 50. Full-MS scans were recorded using the following parameters: FWHM peaks: 15 s, resolution: 140,000 (at m/z 200) AGC-target: 1e6, maximum IT: 200 ms, scan range: m/z 500-2000. Data evaluation was performed using MassMap.

#### RNA analyses

##### RNA isolation and RT-qPCR

RNA was isolated from the cells or tissue samples using the TRIzol reagent (Invitrogen), according to the manufacturer’s protocol. For cells subjected to hypoxia the TRIzol reagent was added while cells were inside the hypoxia workstation. The total RNA was reverse-transcribed into cDNA using M-MuLV Reverse Transcriptase (Thermo Scientific) and random hexamer primers. Quantitative real-time (qRT)-PCR was performed in triplicate, using the SensiMix SYBR Low-Rox One Step kit (Bioline, Cat. No. QT625-05), in a QuantStudio 6 flex cycler (Applied Biosystems). All primer sequences are available upon request.

##### RNaseq analyses

For processing of sequencing data, a customized in-house software pipeline was used. Illumina’s bcl2fastq (v1.8.4) was used to convert the base calls in the per-cycle BCL files to the per-read FASTQ format from raw images. Along with base calling, adaptor trimming and demultiplexing were performed. Quality control of raw sequencing data is performed using FastQC (v 0.11.5). We then map the reads to the mouse transcriptome (Mus_musculus.GRCm38.86). Remaining unmapped reads were then mapped to the mouse genome (mm10). We used rna-STAR (version STAR_2.5.2b) for all the mapping. We allowed no mismatches for the reads < 19b, one mismatch for reads between 20b to 39b, two mismatches for reads between 40b to 59b and so on. We mapped all the reads in the non-splice-junction-aware mode. All other parameters were used in default settings by rna-STAR. The number of aligned reads overlapping it exons for each gene is then counted with the intersection-non-empty mode using htseq-count script available with HTSeq package (version 0.9.1).

##### Differential Expression Analyses

To identify the differentially expressed genes, we first identify unwanted sources of variation (RUVs) and corrected them using RUVSeq (v. 1.8.0). We then used DESeq2 (v. 1.14.1) to perform the differential expression analysis. We then used the genes that were found significantly differentially expressed with base mean > = 35, log2 fold change < > 0.20 and p-adjusted ≤ 0.05.

#### Protein analyses

##### Blue native (BN)–PAGE and respiratory chain activity staining

Standard protocols for BN-PAGE were used as described previously (Mick et al., 2012). Mitochondrial membranes, solubilized in 1% digitonin were subjected for BN-PAGE analysis on a 4%–14% polyacrylamide gradient gel as described (Dekker et al., 1996). Activity staining of native respiratory chain complexes was performed as described previously (Wittig et al., 2006). In brief, complex I activity was visualized in using 2.5 mg/ml nitrotetrazolium blue (NTB) and 0.1 mg/ml NADH, in 5 mM Tris, pH 7.4 at 37°C. Complex IV was visualized, using cytochrome *c* reduced with 15 mg/ml Na-dithionite, which was added to a concentration of 1 mg/ml to the staining solution containing 0.5 mg/ml diaminobenzidine in 50 mM KPi, pH 7.4.

##### Mitochondria isolation and western blotting

Isolation of mitochondria from the cells or cardiac tissues was performed as described in (Lazarou et al., 2009). The tissue was first mechanically disrupted and then homogenized in a potter in homogenization buffer (20 mM HEPES, 70 mM sucrose, 220 mM mannitol, 1 mM EDTA, and 0.5 mM PMSF, pH 7.6). After two spins at 400 x g for 10 min at 4°C to remove cellular debris and nuclear fractions, the mitochondria were separated from the supernatant by centrifugation at 11,000 x g for 10 min at 4°C. Bradford assay was used to determine the protein concentration wherever necessary using RotiQuant (Roth GmbH, Cat No: K015.1). Proteins were separated by SDS-PAGE and transferred to PVDF membranes (Millipore) by semi-dry transfer using standard methods. The membranes were probed with the respective primary antibodies as descried in the reagents section. HRP-coupled secondary antibodies were applied to antigen-antibody complexes and detected by enhanced chemiluminescence on X-ray films.

### Quantification and Statistical Analysis

Data are denoted as mean ± SEM. The exact sample sizes (n) are indicated in the figure legends. The calculation of mean ± SEM and the significance of the difference between groups was performed using Prism 5 software (GraphPad Software, San Diego, CA). The tests used were either unpaired non-parametric or paired non-parametric t tests and ANOVA analysis, unless otherwise noted.

### Data and Software Availability

The Gene Expression Omnibus accession number for the RNA sequencing data reported in this paper is GSE119775. (https://www.ncbi.nlm.nih.gov/geo/query/acc.cgi?acc=GSE119775)

## Supplemental Information

Document S1. Figures S1–S4

Table S1. Expression Levels of Genes under Normoxic and Hypoxic Conditions in Wild-Type MEF Cells, Related to Figure 2

Table S2. Expression Levels of Genes under Normoxic and Hypoxic Conditions in TAZKO MEF Cells, Related to Figure 2

Document S2. Article plus Supplemental Information
