# Supplementary material for: Defective Mitochondrial Cardiolipin Remodeling Dampens HIF-1α Expression in Hypoxia
Source: Cell Rep. 2018 Oct 16;25(3):561–570.e6. doi: 10.1016/j.celrep.2018.09.057 (PMC6205837; doi:10.1016/j.celrep.2018.09.057)
Supplement: Document S1. Figures S1–S4 [file mmc1.pdf]

Cell Reports, Volume *25*

Supplemental Information

Defective Mitochondrial Cardiolipin Remodeling Dampens HIF-1α Expression in Hypoxia

Arpita Chowdhury, Abhishek Aich, Gaurav Jain, Katharina Wozny, Christian Lüchtenborg, Magnus Hartmann, Olaf Bernhard, Martina Balleiniger, Ezzaldin Ahmed Alfar, Anke Zieseniss, Karl Toischer, Kaomei Guan, Silvio O. Rizzoli, Britta Brügger, Andrè Fischer, Dörthe M. Katschinski, Peter Rehling, and Jan Dudek

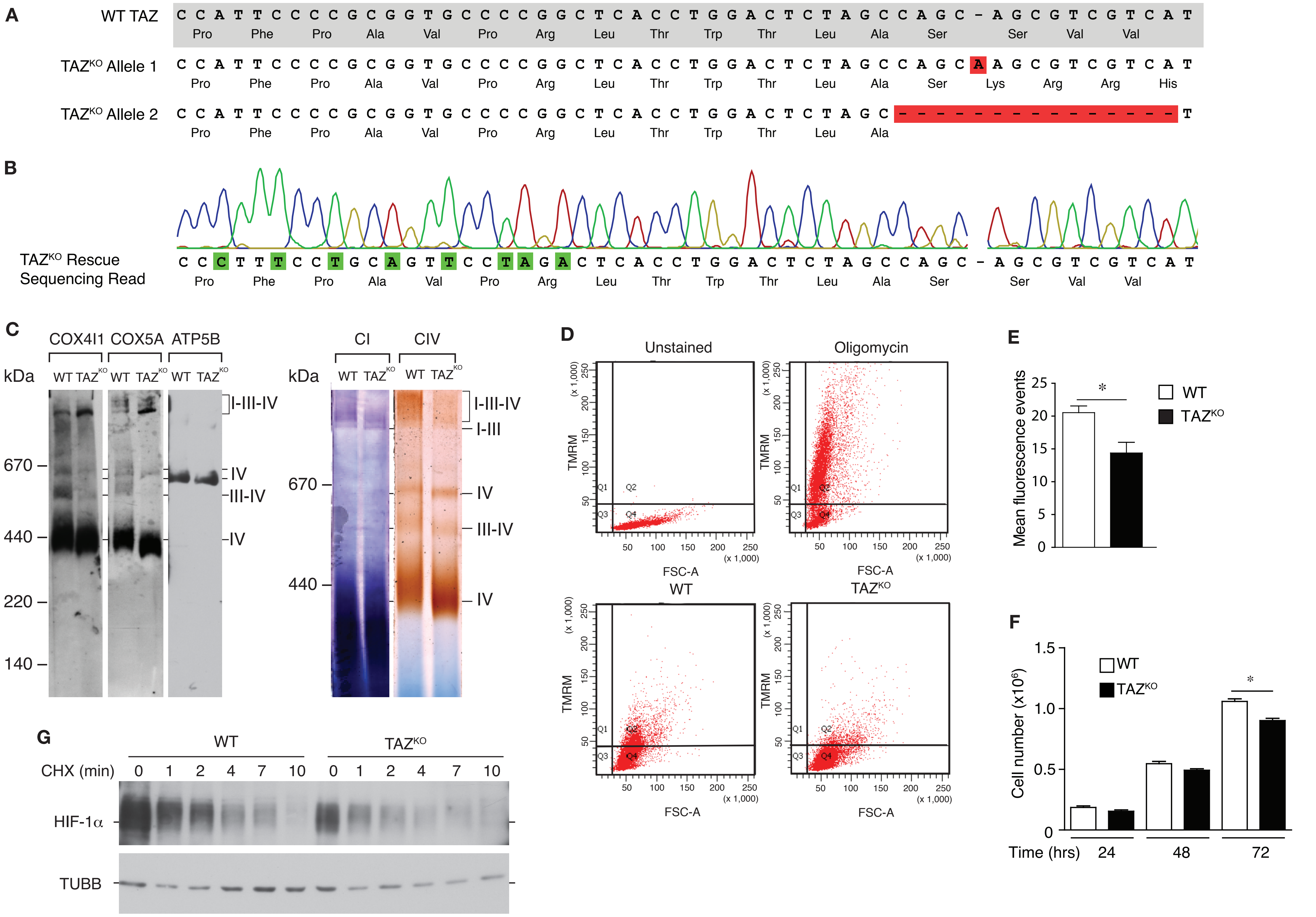
A
WT TAZ
TAZ^KO Allele 1
TAZ^KO Allele 2
B
TAZ^KO Rescue Sequencing Read
C
COX4I1 COX5A ATP5B
kDa
WT TAZ^KO WT TAZ^KO WT TAZ^KO
I-III-IV
IV
III-IV
IV
670
440
220
140
CI CIV
I-III
D
Unstained
Oligomycin
WT
TAZ^KO
TMRM
FSC-A
(x 1,000)
Q1 Q2 Q3 Q4
E
Mean fluorescence events
WT
TAZ^KO
F
Cell number (x10^6)
Time (hrs) 24 48 72
G
CHX (min) 0 1 2 4 7 10 0 1 2 4 7 10
HIF-1α
TUBB

Figure S1. Mitochondrial defects in TAZ^{KO} MEF. Related to Figure 1.

(A) Sequence alignment of WT- Taz locus compared to TAZ^{KO} alleles generated during this study. The mutations in the TAZ^{KO} alleles are indicated in red.

(B) Sequencing read of TAZ^{KO}Rescue clone. Silent mutations introduced during the CRISPR mediated repair process are shown in green.

(C) Mitochondrial membranes, isolated from WT and TAZ^{KO} MEF cells, are solubilized in 1% digitonin and analyzed by BN–PAGE and western blotting with the indicated antibodies (left panel). Respiratory chain complexes were stained for activity of complexes I, and IV (right panel).

(D) Flow cytometry detection of changes in mitochondrial membrane potential ($\Delta\psi$) by staining WT and TAZ^{KO} MEF with TMRM in normoxia compared to unstained cells. WT cells treated with Oligomycin served as positive control. (± SEM, n=3, *p<0.02).

(E) Quantification of the flow cytometric data comparing quadrants Q2 of WT and TAZ^{KO} MEF in supplementary data figure 1D.

(F) Cell counts of WT and TAZ^{KO} MEF cells in glucose containing media (± SEM, n=4, *p<0.0001).

(G) Determination of HIF-1α degradation kinetics in WT and TAZ^{KO} MEF cells using cycloheximide chase for the indicated time points after 24 hours of hypoxia exposure. Equivalent amounts of protein lysates are analyzed via western blotting for anti-HIF-1α and anti-β-tubulin antibodies.

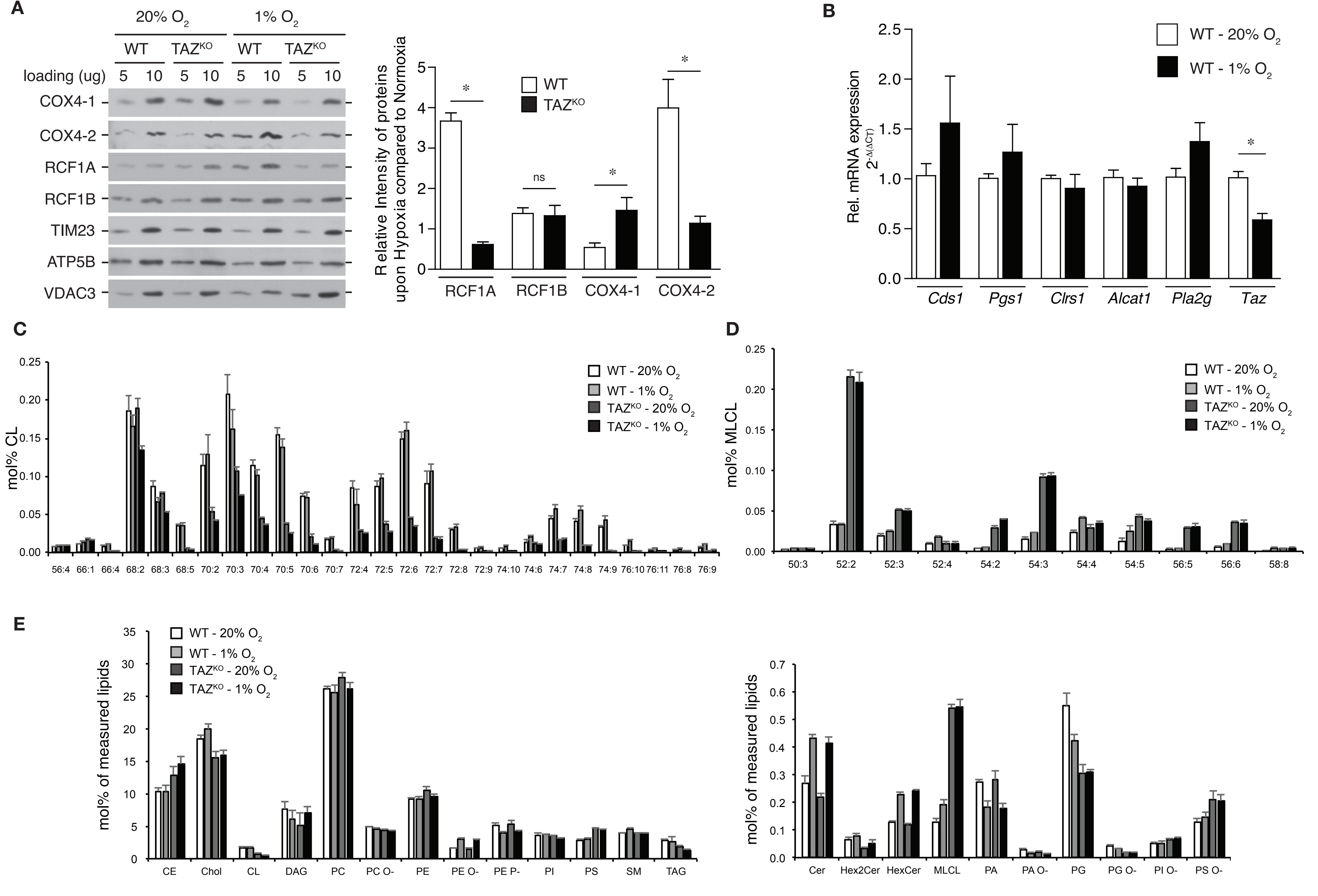

A
20% O2
1% O2
WT
TAZKO
WT
TAZKO
loading (ug) 5 10 5 10 5 10 5 10
COX4-1
COX4-2
RCF1A
RCF1B
TIM23
ATP5B
VDAC3
Relative Intensity of proteins upon Hypoxia compared to Normoxia
WT
TAZKO
ns
RCF1A
RCF1B
COX4-1
COX4-2
B
WT - 20% O2
WT - 1% O2
Rel. mRNA expression 2-Δ(ΔCT)
Cds1
Pgs1
Clrs1
Alcat1
Pla2g
Taz
C
mol% CL
WT - 20% O2
WT - 1% O2
TAZKO - 20% O2
TAZKO - 1% O2
56:4 66:1 66:4 68:2 68:3 68:5 70:2 70:3 70:4 70:5 70:6 70:7 72:4 72:5 72:6 72:7 72:8 72:9 74:10 74:6 74:7 74:8 74:9 76:10 76:11 76:8 76:9
D
mol% MLCL
WT - 20% O2
WT - 1% O2
TAZKO - 20% O2
TAZKO - 1% O2
50:3 52:2 52:3 52:4 54:2 54:3 54:4 54:5 56:5 56:6 58:8
E
mol% of measured lipids
WT - 20% O2
WT - 1% O2
TAZKO - 20% O2
TAZKO - 1% O2
CE Chol CL DAG PC PC O- PE PE O- PE P- PI PS SM TAG
mol% of measured lipids
Cer Hex2Cer HexCer MLCL PA PA O- PG PG O- PI O- PS O-

Figure S2. Differential gene expression profiling in WT and TAZ^{KO} MEF cells under normoxia or hypoxia. Related to Figure 1.

(A) Steady-state protein levels in mitochondria isolated from WT and TAZ^{KO} MEF cells under normoxia or hypoxia for 24 hours, analyzed by western blot with antibodies against indicated proteins (n=3). Graphs on the right indicate the relative intensities of proteins in hypoxia compared to their levels in normoxia (± SEM, n=3, *p<0.05).

(B) qPCR analysis to check for the expression of genes involved in cardiolipin synthesis (*Cds1, Pgs1* and *Clrs1*) and cardiolipin-remodeling *(Alcat1, Taz and Pla2g)* from total mRNA isolated after 24 hours of hypoxia from WT MEF cells. (± SEM, n=3, *p < 0.0049).

(C) Analysis of cardiolipin species isolated from WT and TAZ^{KO} MEF cells after exposure to 24 h hypoxia analyzed by mass spectrometry. (± SEM, n = 4 per genotype).

(D) Analysis of MLCL species isolated from WT and TAZ^{KO} MEF cells after exposure to 24 h hypoxia analyzed by mass spectrometry as described above. (± SEM, n = 4 per genotype).

(E) Analysis of membrane lipids isolated from WT and TAZ^{KO} MEF cells after exposure to 24 h hypoxia analyzed by mass spectrometry. Low abundant lipids are shown in the left panel and high abundant lipids are shown in the right panel(± SEM, n = 4 per genotype).

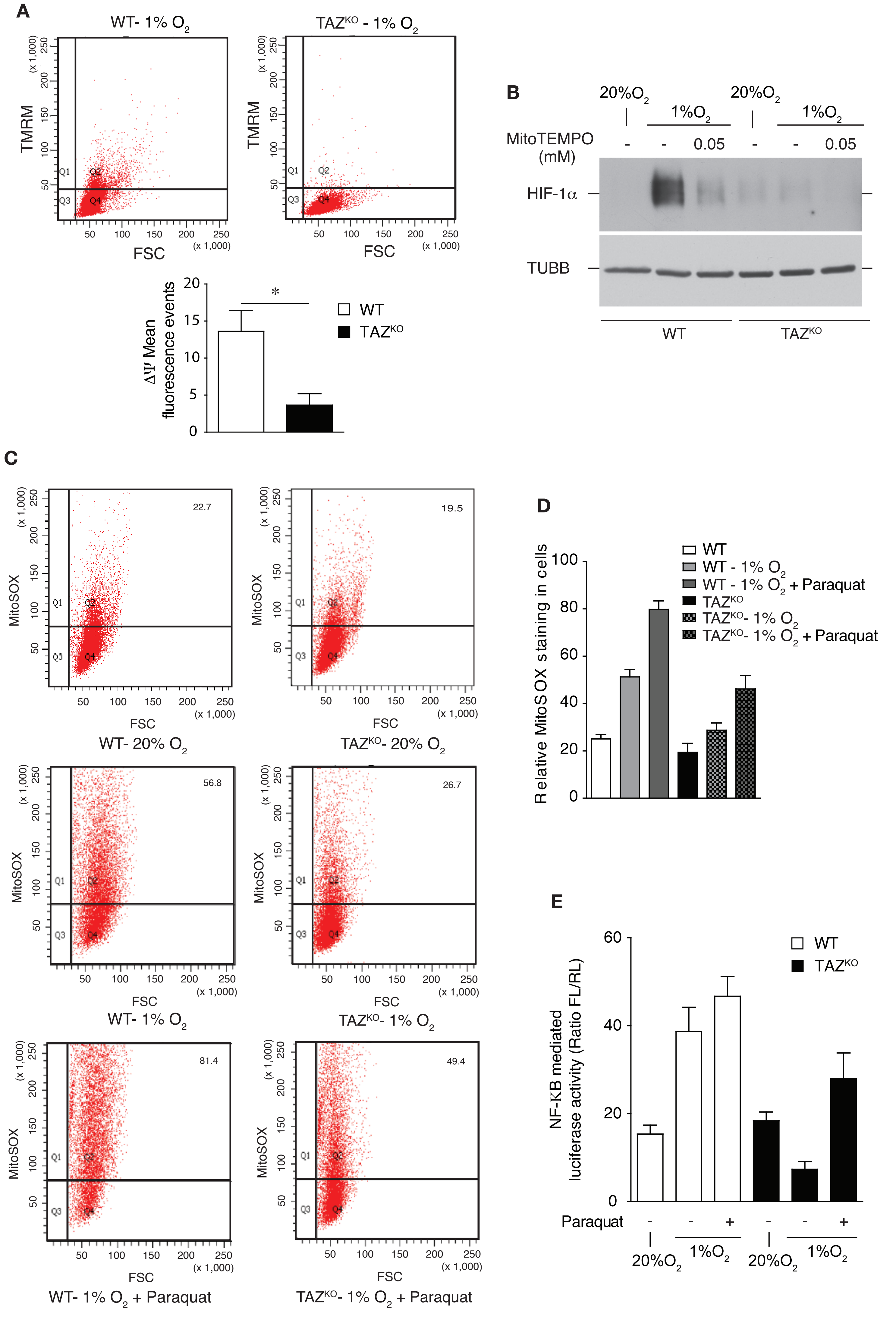

A
WT- 1% O2
TAZKO - 1% O2
TMRM
(x 1,000)
FSC
(x 1,000)
Q1
Q2
Q3
Q4
ΔΨ Mean fluorescence events
*
WT
TAZKO
B
20%O2
1%O2
20%O2
1%O2
MitoTEMPO (mM)
-
-
0.05
-
-
0.05
HIF-1α
TUBB
WT
TAZKO
C
MitoSOX
FSC
22.7
19.5
WT- 20% O2
TAZKO- 20% O2
56.8
26.7
WT- 1% O2
TAZKO- 1% O2
81.4
49.4
WT- 1% O2 + Paraquat
TAZKO- 1% O2 + Paraquat
D
Relative MitoSOX staining in cells
WT
WT - 1% O2
WT - 1% O2 + Paraquat
TAZKO
TAZKO- 1% O2
TAZKO- 1% O2 + Paraquat
E
NF-KB mediated luciferase activity (Ratio FL/RL)
WT
TAZKO
Paraquat
-
-
+
-
-
+
20%O2
1%O2
20%O2
1%O2

Figure S3. Analysis of Effects of ROS in TAZ^{KO} MEF. Related to Figure 3.

(A) Flow cytometric detection of changes in mitochondrial membrane potential ($\Delta\psi$) in WT and TAZ^{KO} MEF exposed to hypoxia and normoxia for 24 hours, assessed by staining with TMRM. The graph below shows the quantification of the flow cytometric data comparing quadrants Q2 of WT and TAZ^{KO} MEF (± SEM, n=3, *$p<0.05$).

(B) Western blot analysis of HIF-1α protein levels under hypoxia in the presence and absence of 0.05 mM MitoTEMPO using anti-β-tubulin antibody as a control

(C) Flow cytometry detection of mitochondrial ROS using MitoSOX in WT and TAZ^{KO} MEF after exposure to hypoxia and normoxia for 24h and treated with paraquat. (± SEM, n=3).

(D) Quantification of mitochondrial ROS in quadrant Q2, measured in Figure S3C.

(E) NF-κB activity measurement using luciferase reporter assay as described in material and methods for 24 hours under normoxia or hypoxia in the presence or absence of paraquat (± SEM, n = 3).

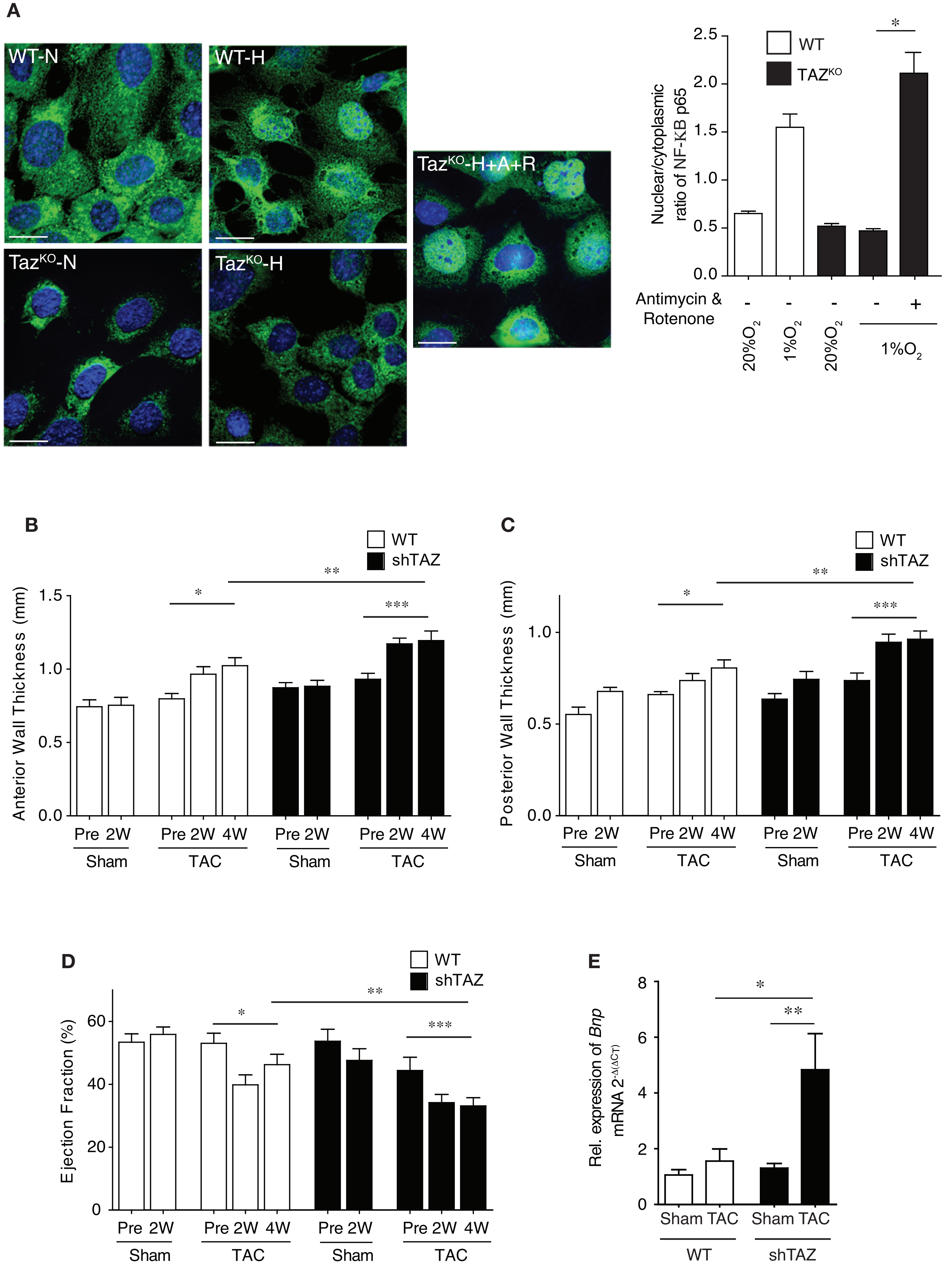
A
WT-N
WT-H
Taz^KO-N
Taz^KO-H
Taz^KO-H+A+R
Nuclear/cytoplasmic ratio of NF-KB p65
2.5
2.0
1.5
1.0
0.5
0.0
WT
TAZ^KO
*
Antimycin & Rotenone
-
-
-
-
+
20%O2
1%O2
20%O2
1%O2
B
WT
shTAZ
Anterior Wall Thickness (mm)
1.5
1.0
0.5
0.0
*
**
***
Pre 2W
Sham
Pre 2W 4W
TAC
Pre 2W
Sham
Pre 2W 4W
TAC
C
WT
shTAZ
Posterior Wall Thickness (mm)
1.0
0.5
0.0
*
**
***
Pre 2W
Sham
Pre 2W 4W
TAC
Pre 2W
Sham
Pre 2W 4W
TAC
D
WT
shTAZ
Ejection Fraction (%)
60
40
20
0
*
**
***
Pre 2W
Sham
Pre 2W 4W
TAC
Pre 2W
Sham
Pre 2W 4W
TAC
E
Rel. expression of Bnp mRNA 2^-Δ(ΔCT)
8
6
4
2
0
*
**
Sham TAC
WT
Sham TAC
shTAZ

Figure S4. Assessment of cardiac function in shTAZ animals after TAC surgery. Related to Figure 3 and 4.

(A) Analysis of NF-κB nuclear translocation in WT and TAZ^{KO} MEF under hypoxia and normoxia in the presence or absence of Antimycin A and Rotenone. NF-κB was detected by immunostaining with antibodies against NF-κB -p65 (green) and Dapi (blue). (Bar=15μm). N denotes: Normoxia; H denotes: Hypoxia; A+R denotes: Antimycin A and Rotenone. The graph on the right shows quantification of cells with nuclear NF-κB to cytoplasmic localization that has been done as described in material and methods (± SEM, n=3, *p<0.05).

(B) Quantification of the anterior wall thickness in WT and shTAZ before (Pre) and after 2 weeks (2W) or 4 weeks (4W) of sham and TAC surgery (± SEM, n=8 in WT, n=12 in shTAZ, ** unpaired t-test: p<0.05, * and *** paired t-test: p<0.05).

(C) Quantification of the posterior wall thickness in WT and shTAZ before (Pre) and after 2 weeks (2W) or 4 weeks (4W) of sham and TAC surgery (± SEM, n=8 in WT, n=12 in shTAZ, ** unpaired t-test: p<0.05, * and *** paired t-test: p<0.05).

(D) Quantification of the percent ejection fraction in WT and shTAZ before (Pre) and after 2 weeks (2W) or 4 weeks (4W) of sham and TAC surgery (± SEM, n=8 in WT, n=12 in shTAZ, ** unpaired t-test: p<0.05, * and *** paired t-test: p<0.05).

(E) qPCR analysis of *Bnp* gene expression from total mRNA isolated from heart samples obtained after 4 weeks of sham and TAC surgery in WT and shTAZ mice (± SEM, n=8 in WT, n=12 in shTAZ, * unpaired t-test: p<0.05, ** paired t-test: p<0.05).
